# Heterogeneity of BCSCs contributes to the metastatic organotropism of breast cancer

**DOI:** 10.1186/s13046-021-02164-6

**Published:** 2021-11-20

**Authors:** Cenzhu Wang, Kun Xu, Runtian Wang, Xin Han, Jinhai Tang, Xiaoxiang Guan

**Affiliations:** 1 Department of Oncology, The First Affiliated Hospital of Nanjing Medical University, 300 Guangzhou Road, Nanjing, Jiangsu 210029 China; 2 Jiangsu Collaborative Innovation Center of Chinese Medicinal Resources Industrialization, School of Medicine & Holistic Integrative Medicine, Nanjing University of Chinese Medicine, Nanjing, Jiangsu 210023 China; 3 Department of General Surgery, The First Affiliated Hospital of Nanjing Medical University, Nanjing, Jiangsu 210029 China

**Keywords:** Breast cancer, Metastatic organotropism, Breast cancer stem cell, Heterogeneity, Seed and soil

## Abstract

Breast cancer is one of the most-common female malignancies with a high risk of relapse and distant metastasis. The distant metastasis of breast cancer exhibits organotropism, including brain, lung, liver and bone. Breast cancer stem cells (BCSCs) are a small population of breast cancer cells with tumor-initiating ability, which participate in regulating distant metastasis in breast cancer. We investigated the heterogeneity of BCSCs according to biomarker status, epithelial or mesenchymal status and other factors. Based on the classical “seed and soil” theory, we explored the effect of BCSCs on the metastatic organotropism in breast cancer at both “seed” and “soil” levels, with BCSCs as the “seed” and BCSCs-related microenvironment as the “soil”. We also summarized current clinical trials, which assessed the safety and efficacy of BCSCs-related therapies. Understanding the role of BCSCs heterogeneity for regulating metastatic organotropism in breast cancer would provide a new insight for the diagnosis and treatment of advanced metastatic breast cancer.

## Background

Breast cancer (BC) is one of the most-common malignant tumors in females worldwide and functions as the leading cause of cancer-related death [1]. In spite of the rapid development of medical technologies, many BC patients still bear the burden of a poor prognosis due to the occurrence of relapse and metastasis. It was reported that 20 - 30 % of BC patients suffered from metastasis after early diagnosis and basic anti-tumor therapies [2]. Moreover, BC patients with metastasis had a remarkably decreased 5-year survival rate of approximately 26 % [3]. However, distant metastasis of BC was recognized to display organotropism, including brain, lung, liver and bone, which represented different symptoms, prognosis and treatments [4]. Bone metastasis was the most frequent BC metastatic event while BC patients with bone metastasis endured bone damages and severe pains, exhibiting the 5-year overall survival (OS) rate of 22.8 %. Lung metastasis with chest tightness or dyspnea displayed the 5-year OS rate of 16.8 %, liver metastasis with emaciation or fatigue showed the 5-year OS rate of 12.5 % while brain metastasis had a worse 5-year OS rate of 12 % with the symptoms of decreased vision, aphasia or balance disorder [5]. Therefore, it is worthy to understand the potential mechanism of the metastatic organotropism in BC, which deserves further investigation.

Breast cancer stem cells (BCSCs) are a small population of breast cancer cells with typical biological features, including self-renewal, multipotent differentiation and tumor-initiating, which play an important role in mediating tumor relapse, metastasis and resistance to chemotherapy or radiotherapy [6]. It was demonstrated that BCSCs exhibited apparent heterogeneity and plasticity, which were of great importance and became a research hotspot in recent years. With regard to heterogeneity, BCSCs can be further classified into different subtypes according to various biological factors, for example biomarkers, epithelial/mesenchymal status and so on. Besides, the plasticity of BCSCs allowed for the reversible transition between different BCSCs subtypes, such as the transition between epithelial and mesenchymal status, which was observed in the process of BC distant metastasis [7].

Most recently, an increasing number of researches have indicated the potential relationship between BCSCs and distant metastasis of BC. BCSCs were found to mediate the process of BC distant metastasis through different biological steps, consisting of stemness maintenance in primary tumor, invading and surviving in blood circulation and colonization in distant organs [8]. However, whether BCSCs also take part in the regulation of metastatic organotropism in BC is still unclear and deserves further investigation. The classical metastatic “seed and soil” theory was proposed in 1889 and clarified the association between tumor cells and host organs [9]. Based on the “seed and soil” theory, we tried to investigate the effect of BCSCs on the metastatic organotropism of BC at both “seed” and “soil” levels, with BCSCs as the “seed” and BCSCs-related microenvironment as the “soil”, which would provide a novel insight for the diagnosis and therapies of advanced metastatic BC patients.

## Heterogeneity of BCSCs

It was reported that BCSCs displayed high heterogeneity among BC patients, which played a significant role in BC recurrence and metastasis, consisting of location in tumor, biological characteristics, tumor-initiating capacity, genetic differences and so on. Based on recent researches, we classified BCSCs into different types, mainly according to their biomarker status, epithelial or mesenchymal status and other biological factors (Table 1).

**Table 1:** Heterogeneity of BCSCs

Heterogeneity		Biological characteristics	Genetic characteristics
Biomarker status	CD24- CD44+ BCSCs	Tumor invasive edge; Highly invasive; [10] Great tumor-initiating capacity:100 cells [11]	Over-expressed genes:IGFBP1, ST8SIA2, PLD5, SCG5, MYOT; KEGG enrichment:focal adhesion, PI3K-AKT signaling [12]
	ALDH+ BCSCs	Center of tumor; Highly proliferative; [10] Great tumor-initiating capacity:500 cells [13]	Over-expressed genes:WNT2,IGF1,DLL1; KEGG enrichment:ribosome, oxidative phosphorylation, proteasome; [12] Mutation:BRCA1 mutation [14]
	CD24- CD44+ & ALDH+ BCSCs	The greatest tumor-initiating capacity:20 cells [13]	-
Epithelial/ mesenchymal status	Epithelial-like BCSCs	Resemble luminal stem cells of normal mammary gland; Identified by ALDH+; Highly proliferative; Mediate colonization of metastatic foci [10]	Up-regulated MET-related genes:CDH1, OCLN, CLDN [15]
	Mesenchymal-like BCSCs	Resemble basal stem cells of normal mammary gland; Identified by CD24- CD44+; Highly invasive; Mediate tumor invasion into blood circulation [10]	Up-regulated EMT-related genes:VIM, ZEB1, ZEB2 [15]

*BCSCs *breast cancer stem cells

### Biomarker status

Classical biomarkers of BCSCs included CD24, CD44 and ALDH1. CD24 is a glycosylated protein connected to the cell membrane, which is responsible for regulating cellular adhesion and metastasis [16]. CD44 is a transmembrane glycoprotein located on cell surface, which can bind various components in extracellular matrix, taking part in cell adhesion, interaction and migration [17]. ALDH1, one member of aldehyde dedydrogenase family, has the ability to oxidize retinol to retinoic acid, participating in regulating self-renewal and maintenance of BCSCs [18].

According to biomarker status, BCSCs can be classified into three types: CD24 - CD44+ BCSCs, ALDH+ BCSCs and BCSCs expressing both CD24- CD44+ and ALDH+. The biological characteristics of three types of BCSCs are various as follows. CD24- CD44+ BCSCs are localized at the tumor invasive edge, staying quiescent with highly invasive characteristics while ALDH+ BCSCs are located at the center of tumor with highly proliferative characteristics. In addition, BCSCs expressing both CD24- CD44+ and ALDH+ are recognized as highly purified BCSCs, exhibiting the greatest tumor-initiating capacity [10]. With regard to the tumor-initiating ability in immune deficient mice, the number of CD24- CD44+ BCSCs was 100 cells, compared with 500 cells in ALDH+ BCSCs, while BCSCs expressing both CD24- CD44+ and ALDH+ phenotypes only needed 20 cells to generate tumors, indicating its most remarkable stemness features [11, 13].

Moreover, it was reported that gene expression signatures varied a lot between CD24- CD44+ and ALDH+ BCSCs groups. The most over-expressed genes contained IGFBP1, ST8SIA2, PLD5, SCG5 and MYOT in CD24- CD44+ BCSCs group, compared with WNT2, IGF1 and DLL1 in ALDH+ BCSCs group. Besides, as demonstrated in KEGG pathways, differentially expressed genes (DEGs) were enriched in focal adhesion and phosphatidylinositol 3-kinase-AKT signaling in CD24- CD44+ BCSCs group while DEGs of ALDH+ BCSCs group were involved in ribosome, oxidative phosphorylation and proteasome [12]. Meanwhile, Heerma van Voss found that BRCA1 mutation could lead to a differentiation block of BCSCs and BRCA1 related BC patients were more likely to have ALDH+ BCSCs [14].

Furthermore, many researches reported that clinicopathological features and survival status showed differences among three type BCSCs. The larger amount of CD24 - CD44+ BCSCs was associated with higher possibility of lymph node metastasis while ALDH+ BCSCs were correlated with microvessel density and estrogen receptor expression [19, 20]. Considering histological types, medullary and metaplastic breast cancers exhibited remarkably increased frequency of BCSCs with CD24- CD44+ and ALDH+ [21]. Besides, BCSCs expressing both CD24- CD44+ and ALDH+ were related with worse progression-free survival (PFS) and could serve as an independent prognostic factor in some subgroups of triple negative breast cancer [22].

### Epithelial/mesenchymal status

It is recognized that the reversible transformation of epithelial cells and mesenchymal cells plays a significant role in regulating the progression of breast cancer. The epithelial - mesenchymal transition (EMT) is defined as the transition from epithelial cells to mesenchymal cells, with reduced cell-cell contacts, loss of polarity and cytoskeleton changes, responsible for enhanced possibility of tumor metastasis, whereas mesenchymal - epithelial transition (MET) exhibits reversible biological behaviors, suggesting high proliferative capacity of tumor cells for colonization in metastatic foci [23].

According to the epithelial or mesenchymal status, BCSCs can be classified into two types: epithelial-like BCSCs and mesenchymal-like BCSCs. Mesenchymal-like BCSCs were characterized as enrichment of EMT-related genes, including VIM, ZEB1 and ZEB2 while upregulation of MET-related genes was discovered in epithelial-like BCSCs, containing CDH1, OCLN and CLDN [15]. In addition, it was demonstrated that epithelial-like and mesenchymal-like BCSCs shared similar biological characteristics separately with luminal and basal stem cells in normal mammary glands. Based on markers CD49f and EPCAM, the heterogeneity of normal mammary gland cells was classified into four types, consisting of EPCAM+ CD49f- epithelial cells, EPCAM+ CD49f+ luminal progenitor cells, EPCAM- CD49f+ stem cells and EPCAM- CD49f- stromal cells. As was reported, EPCAM+ CD49f+ luminal progenitor cells were enriched for epithelial-like BCSCs while EPCAM- CD49f+ stem cells exhibited high proportion of mesenchymal-like BCSCs.Moreover, gene expression profiling indicated that epithelial-like BCSCs could be recognized by expression of ALDH+ while mesenchymal-like BCSCs could be identified via CD24- CD44+ expression in tissue, cell lines and primary xenografts of breast cancer [10].

As is known, the plasticity of BCSCs allowed the reversible transition between epithelial-like and mesenchymal-like status, suggesting the potential function of BCSCs for regulating metastatic behaviors of breast cancer. The matrigel invasion assay indicated that mesenchymal-like BCSCs displayed more invasive properties than epithelial-like BCSCs. According to the experiment results, theories were proposed that mesenchymal-like BCSCs mediated tumor invasion into blood circulation and could resist from anoikis apoptosis whereas epithelial-like BCSCs from niches in distant metastatic organs exhibited high proliferative properties, promoting colonization of metastatic foci [10]. In the meantime, many potential mechanisms were discovered to mediate the plasticity of BCSCs. For example, the lack of miR-200c/141 cluster could promote the generation of mesenchymal-like BCSCs via increasing HIPK1 expression, thus enhancing lung metastasis of breast cancer [24]. Besides, many BCSCs-related signaling pathways were also reported to participate in mediating the process of EMT, thus regulating the metastatic behaviors of breast cancer [25].

### Other factors related with the heterogeneity of BCSCs

Apart from the mentioned factors, BCSCs can also be classified into various types according to other important biological factors. Leth-Larsen R indicated that CD24-CD44+ triple-negative breast cancer cells could be further classified into two types: mesenchymal/basal B and luminal/basal A types. Compared with mesenchymal/basal B type, luminal/basal A type exhibited more typical behaviors of BCSCs, for example mammosphere formation, chemotherapy resistance and so on [26]. In addition, due to alternative splicing, BCSCs marker CD44 was divided into two splice isoforms: CD44 standard splice isoform (CD44s) and CD44 variant splice isoform (CD44v). CD44s was positively associated with the gene signatures of BCSCs while CD44v showed the inverse tendency. Besides, the switching from CD44v to CD44s through splicing factor ESRP1 could promote BCSCs properties [27]. Moreover, Mannello F. reported that a majority of BCSCs displayed marker CD49f and combination of CD24-CD44+ and EpCAM/CD49f could be applied as a novel marker to identify BCSCs subgroups with high mammosphere forming capacity [28].

With regard to BCSCs marker ALDH, 9 out of 19 ALDH isoforms displayed aldehyde dehydrogenase activity with distribution differences. For example, ALDH1A1 was enriched in cytosol and nucleus, ALDH1A3 was found in cytosol while ALDH2 was located in mitochondria [29]. Meanwhile, Vaillant F found that marker CD61/beta3 integrin could recognize a potential BCSCs population with high capability for tumorigenesis in MMTV-wnt-1 tumors [30]. According to Wong NK, in spite of the significance of Notch signaling to BCSCs, BCSCs could still be divided into Notch-dependent and Notch-independent groups. When blocking the Notch signaling, Notch-independent BCSCs group still possessed tumor-initiating capacity [31]. Furthermore, it was proposed by Gyan E that racial heterogeneity of BCSCs played an important role in their effects on clinical outcomes of breast cancer patients. Compared with BCSCs in Caucasians, CD24-CD44+ BCSCs of Asians were explored to significantly influence PFS and OS of breast cancer patients [32].

## Effect of BCSCs on the metastatic organotropism in breast cancer

Despite combination of advanced therapies, many BC patients still possess a worse prognosis due to relapse and metastasis. It is well known that metastatic BC patients always exhibit the organotropism in the process of distant metastasis, including brain, lung, liver and bone. Moreover, metastatic BC patients with different distant metastatic organs always suffer from different symptoms, therapeutic schedules and survival prognosis, which highlights the importance of investigating the underlying mechanism in the organotropism of breast cancer.

Most recently, many researches revealed that there was a potential association between BCSCs and the metastatic organotropism of breast cancer. According to the classical “seed and soil” theory, which was proposed in 1889 to describe the correlation between tumor cells and host organs, we also tried to explore the effect of BCSCs on the organotropism of breast cancer at the “seed” and “soil” levels respectively, with BCSCs as the “seed” and BCSCs-related microenvironment as the “soil”.

### Effect of BCSCs on the metastatic organotropism as “seed”

**Fig. 1:**
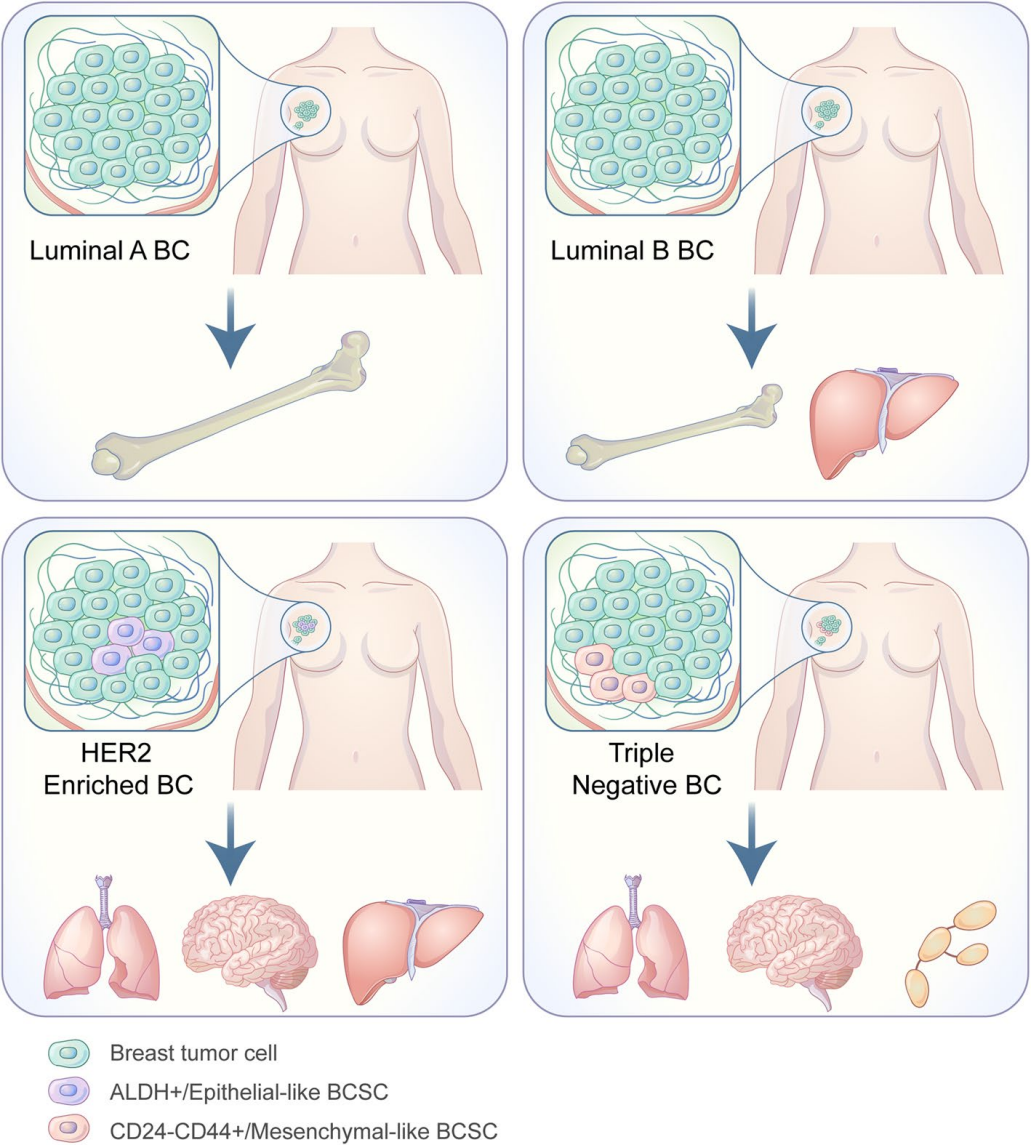
Different molecular subtypes of breast cancer have different metastatic organotropism and BCSCs features. Luminal A breast cancer with a low proportion of BCSCs tends to bone metastasis. Luminal B breast cancer with a low proportion of BCSCs tends to metastasis of bone and liver. HER2 enriched breast cancer with a high proportion of ALDH+/Epithelial-like BCSCs tends to metastasis of lung, brain and liver. Triple negative breast cancer with a high proportion of CD24- CD44+/Mesenchymal-like BCSCs tends to metastasis of lung, brain and distant nodes

It is recognized that BC patients with different molecular subtypes always displayed apparent metastatic organotropism. At the meantime, BC molecular subtypes were reported to be associated with the heterogeneity of BCSCs. Therefore, we propose a hypothesis that the heterogeneity of BCSCs may contribute to the metastatic organotropism in breast cancer, which agrees with the “seed” model of BCSCs and deserves further investigation (Fig. 1) (Table 2).

**Table 2:** Metastatic organotropism and BCSCs features in different molecular subtypes of breast cancer

Molecular subtype	Metastatic organotropism	Features of BCSCs		
		**Proportion**	**Molecular marker**	**Epithelial / Mesenchymal status**
Luminal A	Bone [33]	Low	-	-
Luminal B	Bone, Liver [33, 34]	Low	-	-
HER2 enriched	Lung, Brain, Liver [33]	High	ALDH+ [35–37]	Epithelial-like
Triple negative	Lung, Brain, Distant nodes [33]	High	CD24- CD44+ [35, 38, 39]	Mesenchymal-like [40]

BCSCs: breast cancer stem cells

According to molecular subtypes, breast cancer patients can be classified into four main subgroups, including luminal A, luminal B, human epidermal growth factor receptor 2 (HER2) enriched and triple negative subtypes. Recent researches demonstrated that molecular subtypes of breast cancer are associated with the heterogeneity of BCSCs, consisting of proportion, molecular markers, epithelial or mesenchymal status and so on. As reported by Ricardo S, luminal cell lines displayed high levels of CD24, low levels of CD44 and low ALDH1 activities while HER2-OE cell lines showed enhanced ALDH1 activities and Basal/mesenchymal cell lines had low CD24 expressions and high CD44 expressions [35]. Besides, Xu indicated that basal-like subtype possessed higher CD44 expression with more tendency of epithelial - mesenchymal transition, compared with luminal subtype of breast cancer [40]. Moreover, as to Kong, serum level of CD44 in triple negative subtype was remarkably higher than that in luminal subtype, which could function as an independent prognostic factor in breast cancer [38]. Based on immunohistochemistry analysis of CD24 and CD44 expression in 50 breast cancer patients, Idowu MO also suggested that CD24-CD44+ BCSCs played a significant role in triple negative subtype of breast cancer [39]. In addition, Tsukabe M found that ALDH+ BCSCs were more likely to overlap with HER2-positive tumor cells while luminal A subtype displayed low ALDH1 activities [36]. Similar with Tsukabe M, Park SY discovered that the frequency of ALDH1-positive cells was higher in HER2+ breast tumors than luminal breast tumors [37].

Apart from the association between molecular subtypes of breast cancer and the heterogeneity of BCSCs, many researches also identified the significant correlation between molecular subtypes and the distant metastatic sites in breast cancer, for example brain, lung, liver, bone and lymph nodes. Kennecke H demonstrated that bone served as the most common metastatic site in luminal A and B subtypes whereas the least common metastatic site in basal subtype [33]. At the meantime, Eroles P recognized that luminal A and B subtypes displayed the highest incidence of bone metastasis while luminal B subtype also showed a high rate of liver metastasis [34]. Moreover, compared with luminal A subtype which had the lowest metastatic risk, Kennecke H also indicated that the HER2 enriched subtype showed a higher metastatic rate of lung, brain and liver while the basal-like subtype had a higher metastatic rate of lung, brain and distant nodes. Furthermore, median survival time from first distant metastasis varied a lot among different molecular subtypes of breast cancer, with luminal A patients of 2.2 years, luminal B patients of 1.6 years, HER2 enriched patients of 0.7 year and basal-like patients of 0.5 year [33].

As mentioned above, different molecular subtypes of breast cancer exhibited both heterogeneity of BCSCs and metastatic organotropism of BC and we thus suppose that the heterogeneity of BCSCs may contribute to the selectivity and targeting of distant metastatic organs in breast cancer. The role of BCSCs for mediating the metastatic organotropism of BC is still under research and urges for further investigations.

### Effect of BCSCs-related microenvironment on the metastatic organotropism as “soil”

**Fig. 2:**
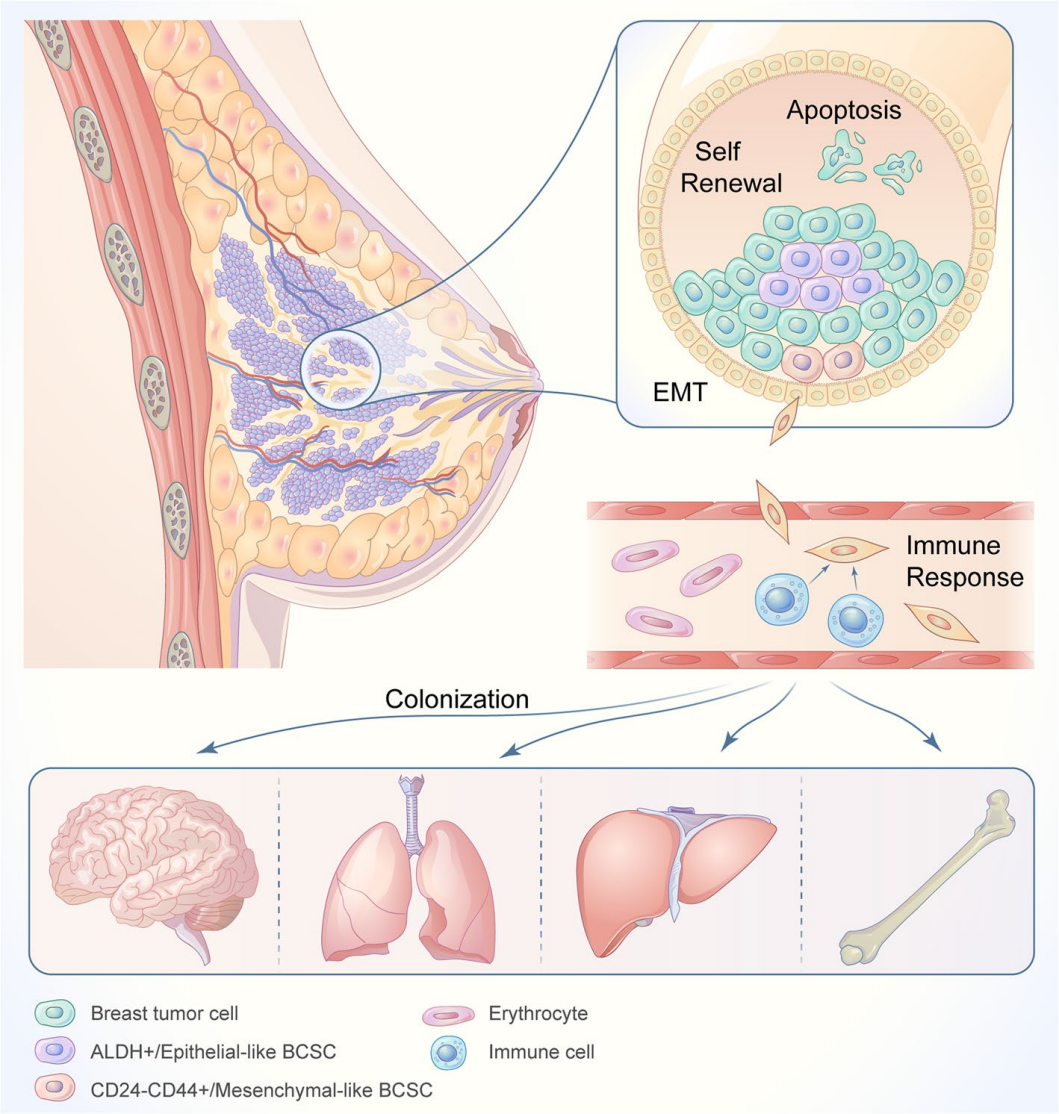
Three important steps of BCSCs-related distant metastasis. BCSCs-related distant metastasis includes three important steps: (1) stemness maintenance in primary tumor; (2) invading and surviving in blood circulation; (3) colonization in distant organs. Stemness maintenance in primary tumor depends on BCSCs self-renewal and apoptosis. Invading and surviving in blood circulation depends on epithelial-mesenchymal transition and escaping from immune response

Apart from the heterogeneity of BCSCs, BCSCs-related microenvironment is also identified to regulate metastatic organotropism of BC, which functions as the “soil”. The BCSCs-related microenvironment is composed of cellular components and non-cellular regulatory factors. Cellular components mainly contain fibroblasts, adipocytes and immune cells while non-cellular regulatory factors consist of extracellular matrix, cytokines, physical and chemical factors. As is known, both of cellular components and non-cellular regulatory factors in BCSCs-related microenvironment can influence the number or function of BCSCs by regulating signaling pathways, suggesting that the interaction between BCSCs and BCSCs-related microenvironment plays an important role in BC progression, including the distant metastasis. As demonstrated by classical theories, BCSCs-related distant metastasis included three important steps: stemness maintenance in primary tumor, invading and surviving in blood circulation and colonization in distant organs. For more details, stemness maintenance in primary tumor depends on biological behaviors, like self-renewal and apoptosis, while EMT and escaping from immune response are responsible for invading and surviving in blood circulation. Hereinafter, we investigated the effect of BCSCs-related microenvironment on the metastatic organotropism in BC (Fig. 2).

#### BCSCs-related Lung metastasis

**Fig. 3:**
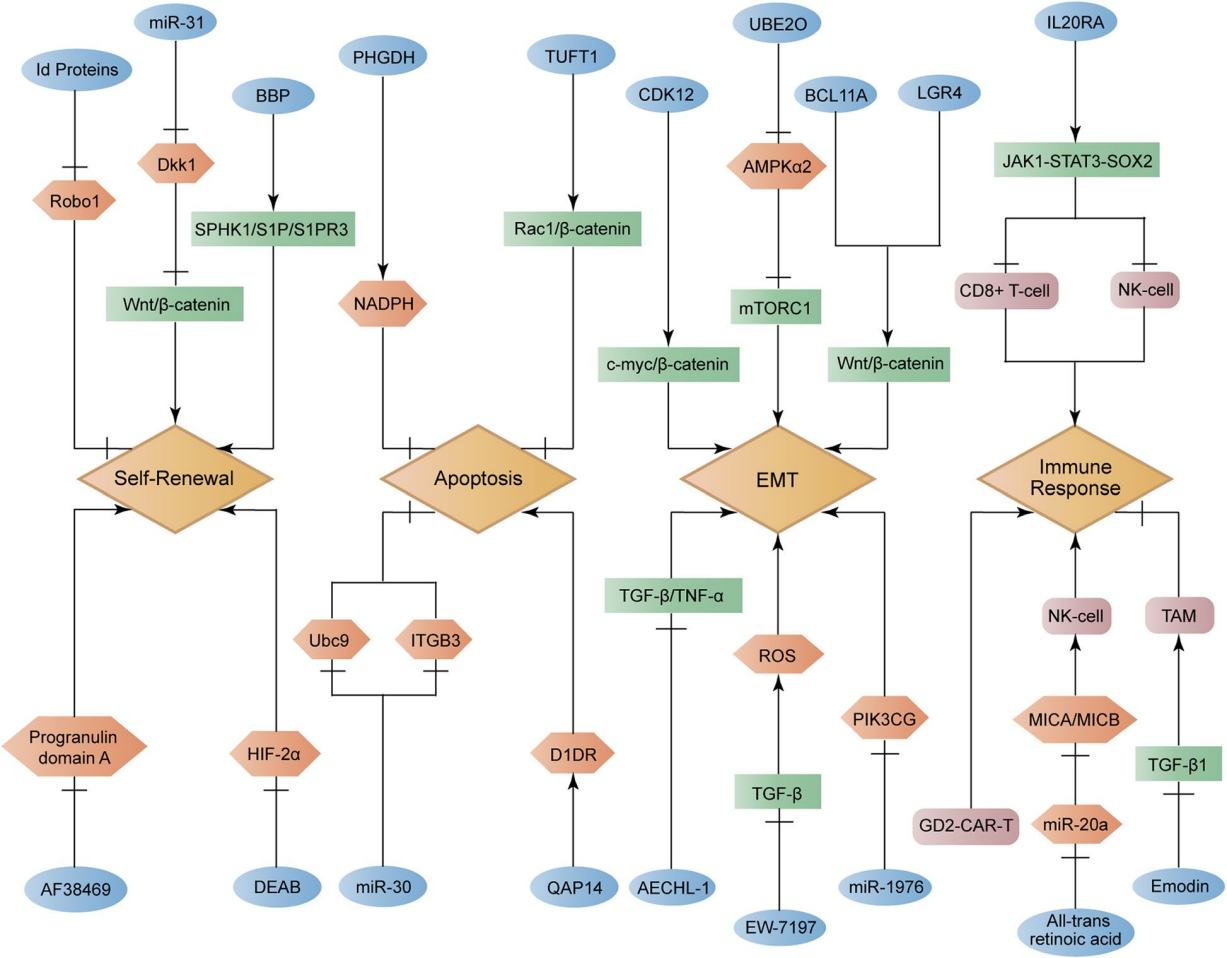
Regulatory networks of BCSCs-related lung metastasis in breast cancer. BCSCs can regulate lung metastasis of breast cancer through various biological processes, including self-renewal, apoptosis, epithelial-mesenchymal transition and immune response. Arrow represents up-regulation while perpendicular line represents down-regulation

We investigated BCSCs-related lung metastasis according to crucial biological behaviors, which participated in BC metastasis, including self-renewal, apoptosis, EMT and immune response (Fig. 3).

The BCSCs self renewal-related lung metastasis was demonstrated to be promoted by miR-31 through inhibiting the Wnt/β-catenin signaling antagonist Dkk1 [41]. Besides, benzyl butyl phthalate (BBP) activated the SPHK1/S1P/S1PR3 signaling and thereby stimulated the BCSCs self renewal-related lung metastasis [42]. Meanwhile, the BCSCs self renewal-related lung metastasis was enhanced by Id proteins via decreasing the expression of Robo1 [43]. On the contrary, AF38469, an orally bioavailable small molecule, was discovered to weaken the BCSCs self renewal-related lung metastasis by down-regulating progranulin domain A [44]. Also, the ALDH inhibitor diethylaminobenzaldehyde (DEAB) displayed a suppressed role in BCSCs self renewal-related lung metastasis by reducing the level of HIF-2α [45].

The BCSCs apoptosis was proclaimed to be suppressed by high expression of PHGDH through up-regulating the level of NADPH, thus stimulating lung metastasis of BC [46]. Besides, the inhibition of BCSCs apoptosis caused by TUFT1 could result from the activation of Rac1/β-catenin signaling pathway, which enhanced BC lung metastasis [47]. However, miR-30 was indicated to work as a promoter in the BCSCs apoptosis via targeting both Ubc9 and ITGB3, thus preventing lung metastasis in BC [48]. In addition, a new oral compound QAP14 was disclosed to increase the expression of dopamine D1 receptor (D1DR), accordingly inducing BCSCs apoptosis and impairing BC lung metastasis[49].

The BCSCs EMT-induced lung metastasis was elucidated to be enhanced by CDK12 via activating the c-myc/β-catenin signaling pathway [50]. Moreover, the important role of Wnt/β-catenin signaling pathway for promoting BCSCs EMT-induced lung metastasis was proven to be supported through both BCL11A and LGR4 [51, 52]. At the meantime, AMPKα2, restrained by UBE2O, exhibited a potential role of weakening mTORC1 signaling pathway and thus accelerated the BCSCs EMT-induced lung metastasis [53]. On the contrary, the reduction of BCSCs EMT-induced lung metastasis was observed to be associated with high level of miR-1976 through targeting PIK3CG [54]. Besides, the TGF-β type I receptor kinase (ALK5) inhibitor EW-7197 served as an inhibitor in BCSCs EMT-induced lung metastasis via impairing the level of paclitaxel-induced reactive oxygen species (ROS) under the regulation of TGF-β signaling pathway [55]. Additionally, the role of AECHL-1, a novel triterpenoid, in repressing BCSCs EMT-induced lung metastasis contributed to its negative regulation of TGF-β/TNF-α [56].

With regard to immune response, it was illustrated that IL20RA could stimulate JAK1-STAT3-SOX2 signaling pathway to suppress recruitment of CD8+ T cells and natural killer cells, which inhibited immune response and thereby enhanced BCSCs-related lung metastasis in BC [57]. Nevertheless, miR-20 functioned as a suppressor in natural killer cell-associated immune response through down-regulating the level of MICA/MICB, thereby enhancing BCSCs-related lung metastasis, which could be restrained by all-trans retinoic acid [58]. Moreover, the natural compound emodin displayed an inhibitor in tumor associated macrophages (TAMs)-related suppressed immune response via blocking the TGF-β1 signaling pathway, which may reduce BCSCs-related lung metastasis in BC [59]. In addition, GD2-targeted chimeric antigen receptor T cells (GD2-CAR-T) was also confirmed to lead to the prevented BCSCs-related lung metastasis [60].

#### BCSCs-related Liver metastasis

**Fig. 4:**
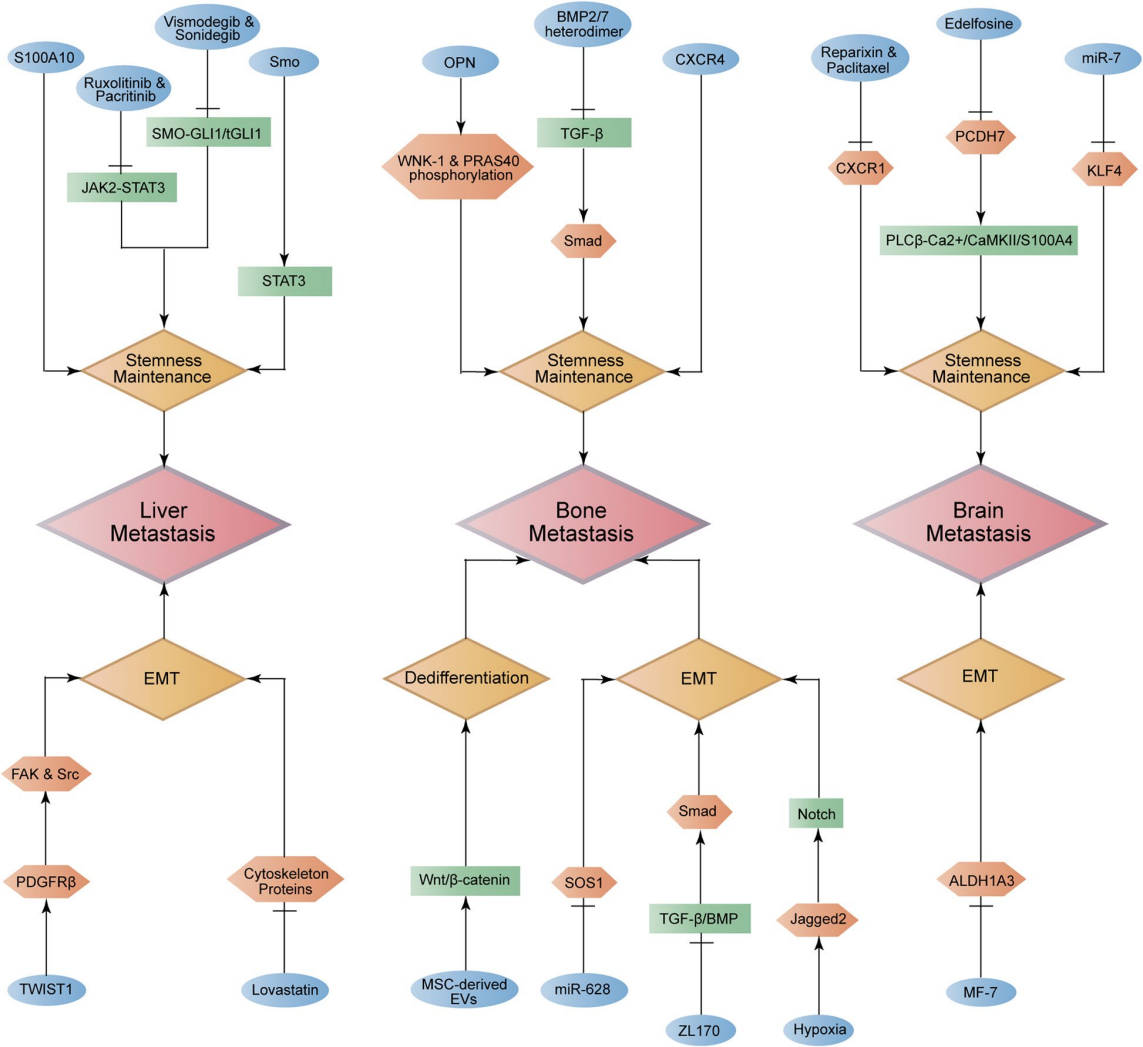
Regulatory networks of BCSCs-related metastasis of liver, bone and brain in breast cancer. The diagram displays the function of BCSCs for regulating breast cancer metastasis of liver, bone and brain through biological processes, like stemness maintenance, epithelial-mesenchymal transition and dedifferentiation. Arrow represents up-regulation while perpendicular line represents down-regulation

We investigated BCSCs-related liver metastasis according to important biological behaviors in BC metastasis, including stemness maintenance and EMT (Fig. 4). It was clarified that smoothened (Smo) up-regulated the level of STAT3, accordingly promoting BCSCs maintenance-related liver metastasis in BC [61]. Besides, S100A10 was discovered to participate in enhancing BCSCs maintenance-related liver metastasis [62]. In addition, combined treatment with JAK2 inhibitors (ruxolitinib and pacritinib) and SMO inhibitors (vismodegib and sonidegib) could served as a suppressor in BCSCs maintenance-related liver metastasis by simultaneously blocking JAK2-STAT3 and SMO-GLI1/tGLI1 signaling pathways [63].

Moreover, it was reported that lovastatin weakened the BCSCs EMT-induced liver metastasis through decreasing the level of cytoskeleton-associated proteins, including FLNA, TMSB10, STMN1 and so on [64]. Also, high level of PDGFRβ, which was stimulated by TWIST1, could increase the expression of FAK and Src, thus inducing BCSCs EMT-induced liver metastasis [65].

#### BCSCs-related bone metastasis

We investigated BCSCs-related bone metastasis according to important biological behaviors in BC metastasis, including stemness maintenance, dedifferentiation and EMT (Fig. 4). It was recognized that bone-derived osteopontin (OPN) supported the BCSCs maintenance-related bone metastasis in BC via enhancing the phosphorylation of WNK-1 and PRAS40 [66]. Meanwhile, TGF-β, which was inhibited by BMP2/7 heterodimer, could strengthen the BCSCs maintenance-related bone metastasis through activating the level of Smad [67]. Additionally, high expression of CXCR4 was found to be associated with the enhanced BCSCs maintenance-related bone metastasis[68].

At the meantime, mesenchymal stem cell (MSC)-derived extracellular vesicles (EVs) were announced to strengthen the Wnt/β-catenin signaling pathway, which promoted the dedifferentiation of breast cancer cells into BCSCs and enhanced colonization of BC in bone marrow[69]. What’s more, hypoxia-induced high expression of Jagged2 was reported to stimulate the Notch signaling pathway,thus increasing BCSCs EMT-induced bone metastasis[70]. Moreover, a natural small-molecule compound ZL170 was investigated to refrain TGF-β/BMP, which enhanced BCSCs EMT-induced bone metastasis via up-regulating Smads[71]. Furthermore, it was clarified that miR-628 could act as a suppressor in BCSCs EMT-induced bone metastasis through targeting SOS1 [72].

#### BCSCs-related brain metastasis

We investigated BCSCs-related brain metastasis according to important biological behaviors in BC metastasis, including stemness maintenance and EMT (Fig. 4). It was confirmed that the role of miR-7 in impairing the BCSCs maintenance-related brain metastasis contributed to its negative regulation of KLF4 [73]. Besides, PCDH7 was illustrated to be refrained by the selective PLC inhibitor edelfosine and showed a potential of supporting BCSCs maintenance-related brain metastasis by stimulating the PLCβ-Ca^2+^/CaMKII/S100A4 signaling pathway [74]. Moreover, combination of reparixin and paclitaxel was recognized to suppress BCSCs maintenance-related brain metastasis via decreasing the level of CXCR1 [75]. However, the ALDH1A3 inhibitor MF-7 was elucidated to weaken BCSCs EMT-induced brain metastasis by impairing the expression of ALDH1A3 [76].

### Current situation and future prospects

As mentioned above, we investigated the association between BCSCs and BC metastatic organotropism at the “seed” and “soil” levels, with BCSCs as the “seed” and BCSCs-related microenvironment as the “soil”. The heterogeneity of BCSCs could contribute to BC metastatic organotropism while BCSCs-related microenvironment regulated BC metastatic organotropism through different signaling pathways in the process of BCSC self-renewal, apoptosis, EMT and immune response.

With regard to signaling pathways, some classical signaling pathways have been widely recognized in BCSCs regulation, consisting of Wnt, Notch and Hedgehog signaling pathways. These classical signaling pathways were also reported in BCSCs-related BC metastatic organotropism, which deserved close attentions, including the miR-31-Dkk1-Wnt/β-catenin axis, the BCL11A-Wnt/β-catenin axis, the LGR4-Wnt/β-catenin axis in BC lung metastasis, the Vismodegib&Sonidegib-SMO-GLI1/tGLI1 axis in BC liver metastasis and the MSC-derived EVs-Wnt/β-catenin axis, the hypoxia-Jagged2-Notch axis in BC bone metastasis. Apart from classical signaling pathways, an increasing number of brand-new signaling pathways were also discovered and worthy of further exploration.

However, there are still some limitations in current researches. On the one hand, current researches for BCSCs-related BC metastatic organotropism remain superficial and lack of further investigations for underlying molecular mechanisms. According to current experimental researches, we would like to put forward potential hypotheses for BCSCs-related BC metastatic organotropism, for example cross-talk between BCSCs and distant organs, pre-metastatic niche formation in distant organs and so on, which need for exploration in the future. On the other hand, current research results mainly come from cell and mice experiments, whose guiding value for clinical diagnosis and treatment of BC patients remains unclear. Therefore, it is of great significance to perform clinical trials for verifying the anti-tumor efficacy and safety of potential BCSCs-related targets and signaling pathways.

## Clinical trials of BCSCs-related therapies

Despite traditional therapies of surgery, chemotherapy, radiotherapy, immunotherapy and so on, a majority of BC patients still suffer from distant metastasis, which is believed to be driven by BCSCs. Therefore, an increasing number of researchers begin to focus on BCSCs-related therapies to overcome clinical challenges. Most recently, a variety of clinical trials have been conducted to investigate the safety and efficacy of BCSCs-related therapies. Current BCSCs-related clinical trials are mainly designed to target receptors of BCSCs or classical signaling pathways of BCSCs regulation, including Notch signaling pathway, Hedgehog signaling pathway and Wnt signaling pathway, which are performed in breast cancer patients or advanced solid tumor patients with partial breast cancer patients (Table 3).

**Table 3:** Clinical trials of BCSCs-related therapies

Modality	Clinical trial	Phase	Study arms	Enrolled population	Patients (n)	Status	Preliminary antitumor efficacy
Monotherapy	NCT00106145 [77]	I	MK-0752	adult patients with advanced solid tumors	Total: 103 BC: 24	Completed	Objective CR: 1 glioma patient SD ≥4 months: 10 glioma patients BC patients: no significant efficacy
Combination therapy	MK-8669-049 NCT01295632 [78]	I	MK-0752 + Ridaforolimus	advanced solid tumors	Total: 30 BC: 2	Completed	CR: 1 HNSCC patient PR: 1 HNSCC patient SD ≥6 months: 1 HNSCC patient BC patients: no significant efficacy
Combination therapy	NCT00645333 [79]	Ib	MK-0752 + Docetaxel	locally advanced or metastatic breast cancer	30	Completed	PR: 11 patients SD: 9 patients PD: 3 patients
Combination therapy	NCT02784795 [80]	Ib	LY3039478 + taladegib LY3039478 + LY3023414 LY3039478 + abemaciclib	advanced or metastatic solid tumors	Total: 63 BC: 12	Completed	CR/PR: none DCR: Part A: NA, Part B: 18.8 %, Part C: 26.1 % BC patients: no significant efficacy
Combination therapy	EDALINE NCT02027376 [81]	Ib	sonidegib (LDE225) + docetaxel	triple negative advanced breast cancer	12	Completed	Median TTP: 42.5 days (95 % CI: 29-155 days)
Monotherapy	NCT01351103 [82]	I	WNT974	advanced solid tumors	Total: 94 BC: 20	Recruiting	SD: 16 % BC patients: no significant efficacy
Monotherapy	NCT01861054 [83]	II	Reparixin	operable HER2-negative breast cancer	20	Terminated	BCSC markers CD24-/CD44+ and ALDH+: decrease ≥20 %
Combination therapy	NCT02001974 [84]	Ib	Reparixin + Paclitaxel	HER2-negative metastatic breast cancer	30	Completed	RR: 30 % DR >12 months: 2 patients

*CR *complete response, *PR *partial response, *SD *stable disease, *DCR *disease control rate, *TTP *time to progression, *RR *response rate, *DR *durable response, *HNSCC *head and neck squamous cell carcinoma, *BC *breast cancer, *NA *not available

MK-0752 is an oral inhibitor of γ-secretase, which could function as an enzyme for activating Notch pathway, suggesting that applying MK-0752 to block Notch pathway could prevent the progress of BCSCs. Recently, a phase I clinical trial was conducted to investigate the safety and anti-tumor efficacy of MK-0752 in 103 adult patients with advanced solid tumors, including 24 breast cancer patients. With regard to pharmacokinetics and pharmacodynamics, MK-0752 had a half-life of approximately 15 h while it exhibited remarkable role of inhibiting Notch pathway with 1800 mg to 4200 mg weekly dose levels. Among glioma patients, 1 patient had an objective complete response (CR) and 10 patients had a stable disease (SD) longer than four months whereas breast cancer patients showed no significant efficacy. Besides, the most common drug-related toxicities lied in gastrointestinal symptoms and fatigue [77]. MK-8669-049 is another phase I study to explore the combination of MK-0752 and mTOR inhibitor ridaforolimus in 30 advanced solid tumor patients, including 2 breast cancer patients. Primary results indicated that patients suffered from grade 2 or 3 dose-limiting toxicities (DLT) among 20 mg or 30 mg ridaforolimus groups while maximum tolerated dose (MTD) lied in 20 mg daily ridaforolimus 5 days/week + 1800 mg weekly MK-0752. As to anti-tumor efficacy, among head and neck squamous cell carcinoma (HNSCC) patients, 1 patient had a CR, 1 patient had a partial response (PR) and 1 patient had a SD longer than six months whereas breast cancer patients showed no significant efficacy [78]. Another phase Ib clinical trial was performed to integrate MK-0752 and docetaxel in 30 locally advanced or metastatic breast cancer patients. The analysis data informed that specific DLTs included pneumonitis, hand-foot syndrome, LFT elevation and diarrhea while 11 patients had a PR, 9 patients had a SD and 3 patients had a progressive disease (PD) [79]. Meanwhile, crenigacestat (LY3039478) is another potential Notch inhibitor, whose combination with other anti-tumor drugs could play a synergistic role for inhibiting BCSCs. A phase Ib clinical study was conducted to explore the combination of LY3039478 with other anti-tumor target agents (taladegib, LY3023414 or abemaciclib) in 63 patients with advanced or metastatic solid tumors, including 12 breast cancer patients. As shown in this trial, DLTs included diarrhea, nausea and vomiting, which occurred in 12 patients while 75.0-82.6 % adverse events were beyond Grade 3. Besides, the MTD of patients with LY3023414 was 25 mg compared with 50 mg among patients with abemaciclib while disease control rate exhibited 18.8 % or 26.1 % among patients with LY3023414 or abemaciclib. However, no CR or PR was observed and breast cancer patients showed no significant efficacy [80].

Moreover, Smo plays an important role in regulating Hedgehog signaling pathway, which is associated with the progress of BCSCs, while sonidegib (LDE225) serves as a selective oral inhibitor of smo. EDALINE is a phase Ib clinical trial to investigate the combination of sonidegib (LDE225) and docetaxel in 12 triple negative advanced breast cancer patients. The latest results informed that no pharmacokinetic interactions exhibited between sonidegib and docetaxel while patients with 800 mg sonidegib had grade 3 adverse events, including neutropenia, CPK increase, leukopenia and paresthesia. Besides, the addition of LDE225 plus docetaxel could lead to a median time to progression (TTP) of 42.5 days [81]. In addition, porcupine is a membrane-bound O-acyltransferase enzyme, participating in regulating Wnt signaling pathway, while WNT974 is a potential inhibitor of porcupine. A phase I clinical study using WNT974 was performed in 94 patients with advanced solid tumors, including 20 breast cancer patients. Preliminary results showed that recommended dose for expansion was 10 mg once daily while adverse events lied in dysgeusia. Besides, as to antitumor efficacy, 16 % of patients had a SD whereas breast cancer patients showed no significant efficacy [82]. At the meantime, chemokine receptor 1 (CXCR1) is one of the receptors selectively expressed in BCSCs. Reparixin is an investigational allosteric inhibitor of CXCR1, indicating its potential role of reducing BCSCs. Most recently, a phase II study of reparixin has recruited 20 operable Her2-negative breast cancer patients to assess the safety and anti-tumor efficacy. As shown in this trial, BCSCs markers CD24-/CD44+ and ALDH+ decreased by more than 20 % in part of patients, suggesting the potential role of reparixin for reducing BCSCs [83]. Another phase Ib trial that integrated reparixin with paclitaxel was designed in 30 Her2-negative metastatic breast cancer patients. The update results demonstrated that no pharmacokinetic interactions exhibited between reparixin and paclitaxel while 2 patients had durable response more than 12 months and the response rate (RR) reached 30 % [84].

## Conclusions

Breast cancer is one of the leading female malignant tumors with a high risk of relapse and distant metastasis. BC patients with distant metastasis always exhibit apparent organotropism, including brain, lung, liver and bone. BCSCs are a small population of breast cancer cells with tumor-initiating capacity, which participate in regulating distant metastasis of BC. However, whether BCSCs have an effect on the metastatic organotropism of BC is still unclear and deserves further investigation. In this review, we firstly investigated the heterogeneity of BCSCs according to biomarker status, epithelial or mesenchymal status and other biological factors. Then, we explored the effect of BCSCs on the BC metastatic organotropism based on the “seed and soil” theory, with BCSCs as the “seed” and BCSCs-related microenvironment as the “soil”. At last, we summarized clinical trials which assessed the safety and efficacy of BCSCs-related therapies. Exploring the potential correlation between BCSCs and the metastatic organotropism of BC is of great significance and provides guidance for advanced metastatic breast cancer.

## Data Availability

Not applicable.

## Abbreviations

BBP: Benzyl butyl phthalate
BC: Breast cancer
BCSCs: Breast cancer stem cells
CD44s: CD44 standard splice isoform
CD44v: CD44 variant splice isoform
CR: Complete response
CXCR1: Chemokine receptor 1
D1DR: Dopamine D1 receptor
DEAB: Diethylaminobenzaldehyde
DEGs: Differentially expressed genes
DLT: Dose-limiting toxicities
EMT: Epithelial - mesenchymal transition
EVs: Extracellular vesicles
GD2-CAR-T: GD2-targeted chimeric antigen receptor T cells
HER2: Human epidermal growth factor receptor 2
HNSCC: Head and neck squamous cell carcinoma
MET: Mesenchymal - epithelial transition
MSC: Mesenchymal stem cell
MTD: Maximum tolerated dose
OPN: Osteopontin
OS: Overall survival
PD: Progressive disease
PFS: Progression-free survival
PR: Partial response
ROS: Reactive oxygen species
RR: Response rate
SD: Stable disease
Smo: Smoothened
TAMs: Tumor associated macrophages
TTP: Time to progression
